# Evidence-Based Reconstruction of Memories of a Disdainful Hookup: Identifying Risk Factors and Preventing Further Victimization

**DOI:** 10.3390/bs14050367

**Published:** 2024-04-26

**Authors:** Lidia Puigvert, Ramon Flecha, Sandra Racionero-Plaza

**Affiliations:** Department of Sociology, University of Barcelona, 08034 Barcelona, Spain; lidia.puigvert@ub.edu (L.P.); ramon.flecha@ub.edu (R.F.)

**Keywords:** coerced forgetting, gender violence, autobiographical memory, disdainful hookups, memory reconstruction

## Abstract

Autobiographical memories of close relationships have been shown to have strong influence in health and life. Yet, there is no research published about longitudinal memory reconstruction of violent sporadic relationships while reading and discussing scientific evidence on gender violence victimization. This article presents a novel case of the reconstruction throughout time of the memory of a disdainful hookup experienced by a young woman. The victim’s diary and an interview were the sources of data collection. The analytical categories were developed in dialogue with the participant. The results indicate that, as the subject learned scientific evidence on gender violence in sporadic relationships, she progressively recalled details of the episode that she had self-censored before, became aware of the very violent nature of the hookup, rejected the relationship, and freed her desire for satisfactory romantic relationships.

## 1. Introduction

The scientific literature has clarified the influence of first sexual-affective experiences on subsequent sexual-affective relationships [1,2,3,4]. Such first sexual-affective episodes, today, are mostly sporadic relationships [5]. Whereas positive intimate relationships establish a safe emotional base and a positive framework for later relationships [6], toxic sexual relationships have proven to worsen socio-emotional development and physical and mental health [7,8,9,10,11]. Among these toxic relationships, hookups have been identified as a context with a high prevalence of sexual violence [5,12]. Research in the field of gender violence victimization has shown that nightlife settings, such as discos and bars, are socializing contexts where violent sporadic relationships are the rule [13]. Other research has shared that episodes of violent sporadic relationships worsen physical and mental health [14], which has led to the consideration of disdainful hookups as a powerful social determinant of health [15].

Research about gender violence has indicated that the coercive dominant discourse is one reason behind the increasing number of women who are victims of violent sporadic sexual-affective relationships [5,16]. Such discourse presents men who have aggressive attitudes and behaviors as most sexually attractive [1]. Studies have shown that such men are preferred for hookups more than for stable relationships [17]. The coercive dominant discourse is boosted and reproduced in interactions in the peer group [17], with some female friends pushing others to engage in such kinds of hookups, presenting them as “cool”, while sexual-affective relationships that are egalitarian are presented as boring.

Such pressure can also translate into a storytelling of the violent sexual experience that does not correspond with what actually occurred but that adapts to what the audience, the peer group, pushes the young woman to explain and what they want to hear, which is that the sexual experience was sexually satisfying and exciting, when research says the opposite [17]. This phenomenon of reconstruction and falsification can be explained by the influence of social interactions (also influenced by the coercive dominant discourse) in shaping autobiographical memories [18,19,20]. Given the prospective functions of autobiographical memories [21,22], how episodes of sexual violence in hookups are stored in the young woman’s memory is central. A memory that is imprisoned by the coercive dominant discourse would keep the violent episode as attractive or cool [17], and such memory might well impel the young woman to project future sexual-affective relationships along similar lines and even to shape her future sexual-affective relationships similarly. If this occurs, the young woman is prone to revictimization.

Memory researchers have acknowledged that autobiographical memories of traumatic and violent experiences are malleable via communication [18,19]. Other studies have focused on interventions such as the writing of a diary about voluntary and involuntary memories of an autobiographical event by individuals with post-traumatic stress disorder [23]. Clinical studies of subjects with histories of traumatic experiences, such as child sexual abuse or war veterans, have indicated how dialogue-based therapeutic interventions around the memory of the trauma may be beneficial. Benefits include improved health and wellbeing [7,8,9,10,11,24]. In addition, there is scientific literature pointing out that the avoidance of autobiographical memories may hinder the solution of some problems, such as the repulsive body image in some women, when such image is stored in autobiographical memories related to their bodies [25]. Based on this evidence, as well as considering that other social experiences such as reading [26] can lead to free and voluntary memory recall and reconstruction, this article reports research addressing the question of whether and how a young woman who had experienced a sporadic sexual-affective relationship self-revised and self-reconstructed her memories of the hookup while she was learning the scientific evidence on social pressure to have disdainful hooking ups and discussing this evidence. A particular focus was placed on exploring whether the experience of reading and discussing scientific evidence supported a reconstruction not enslaved to the dominant coercive discourse. This process has been defined as “evidence-based reconstruction of memory” and refers to memory reconstruction promoted by learning and dialogue about scientific evidence related to the semantic and emotional content of the episode. The exploration reported here is novel in the field of autobiographical memory research and can suggest new approaches to be investigated for preventing gender-based violence victimization in adolescence and youth, a social problem of worldwide concern.

## 2. Materials and Methods

The study was conducted with the communicative methodology of research, which has created the concepts of co-creation and social impact. Both concepts have now become requirements of Horizon Europe, the Framework Programme of Research of the European Union, for research in all sciences. One of the main characteristics of the communicative methodology of research is including scientific evidence of social impact in the dialogue with participants regarding the topic being studied. In the case of our research, the process started when the participant victim discovered the scientific evidence of social impact about disdainful hookups. She decided to start a dialogue with one researcher in this field. All the dialogues were initiated by the victim, while the researcher provided the scientific evidence the participant wondered about at each moment. When the victim became a survivor, she asked the researcher with whom she maintained this dialogue to write a scientific publication about her case of evidence-based memory reconstruction in order to provide new evidence that could be helpful to other victims of the same or similar experiences and to therapists working with these victims.

### 2.1. Case History and Investigation

Simonetta is a heterosexual woman living in Southern Europe. She is in her twenties, holds a bachelor’s and master’s degree, and is from upper-middle-class background. At the time of the study, Simonetta reported no memory conditions. She reported to have had a disdainful sporadic relationship when she was 18. This was a one-night, violent sexual episode that started in a disco and finished right outside that setting. This episode happened days after she turned 18. The relationship lasted for five minutes. A year later, when Simonetta entered college, she discovered scientific publications with evidence regarding the dominant coercive discourse and its influence in promoting violent sporadic sexual-affective relationships. She has been reading and having conversations about this scientific evidence since then.

We report the results of an in-depth analysis of Simonetta’s memories of her disdainful hookup. The analysis sought to investigate the changes in Simonetta’s autobiographical memory throughout time by comparing 10 recalls at different time points and including the scientific evidence that she learned (via reading and dialogue) before or in between each of the recalls.

The study was conducted in accordance with the Declaration of Helsinki and was evaluated and approved by an Ethics Research Committee. After obtaining the participant’s written informed consent, an interview was conducted to learn about the scientific evidence Simonetta read and had conversations about before the different recalls as well as to identify her perceptions on how this process of evidence-based reconstruction of memory has helped her at present, if at all. Simonetta’s memories of the event as they were written in her diary were also analyzed. The diary included both the original writing of the story at the time when it occurred as well as 10 later recalls.

As informed by the participant, the writings of the recalls in the subject’s diary occurred mostly in her house, with the subject always being alone. The subject gave the diary to the research team once she had requested the researcher who she has asked for scientific evidence to write a scientific article on her case. The diary was later analyzed by the research team in a university research setting.

Simonetta learned scientific evidence via reading scientific articles and having conversations about them. As explained by Simonetta, these conversations were with people that she freely chose. In some cases, these individuals were researchers and, in other cases, female friends to whom she forwarded the scientific articles she had read. Simonetta shared with the researchers that every time she learned about new evidence, she recalled the episode in new ways; mostly, she recalled new details.

The timings of the episodes analyzed as well as the timing of each recall are presented in Table 1. Simonetta explained that she had always written these memories in solitude, and no one had ever seen them until this research.

An in-depth interview was conducted with a communicative orientation [27]. This meant that, beyond the questions planned by the research team, the interviewer welcomed other topical reflections from the participant. The interview was held in the university setting. In this interview, Simonetta mostly shared how the evidence-based reconstruction of the memory of the disdainful hookup had enhanced both her current health and relationships. The interview was audio-recorded and later transcribed.

Development of analytical categories: The researchers developed an initial short list of categories derived from the literature on preventive socialization of gender violence, in particular, literature on the dominant coercive discourse and disdainful hookups. This list also included categories informed by the memory literature, particularly that on autobiographical memory [18,22] and the social turn in memory research [19]. In line with the communicative methodology of research, every category was shared with Simonetta to ultimately revise the categories together. As a result, the list of categories was updated; some categories were kept as they were initially, other ones were modified, and other ones were newly developed.

### 2.2. Analysis of the Autobiographical Memory of the Event

After the dialogue with the participant regarding the categories, eleven were developed: forced sex; consciousness of being despised; consciousness of absence of pleasure; consciousness of falsification of memory; peer coercion by young women; enslaving oneself to peer coercion; consciousness of deterioration; consciousness of coerced forgetting; recalling egalitarian men, their value, and attractiveness; consciousness of devaluation; and incoherence. The categories are defined as follows.

*Forced sex* is understood as non-consensual sex and therefore includes forcing one person to sexual activity that she does not want to, even using physical force. *Consciousness of being despised* means instances where the person shows awareness of being despised and humiliated in the sexual relationship. *Consciousness of absence of pleasure* refers to indicators of the person’s awareness of absence of sexual pleasure in the relationship and even feeling harm. *Consciousness of falsification of memory* means acknowledgement of conscious falsification of the memory. *Peer coercion by young women* is the process in which young women who are peers coerce another girl in the group to engage in violent sporadic sexual relationships. *Enslaving oneself to peer coercion* implies the person conforming to the mandates of peers regarding having violent hookups. *Consciousness of deterioration of health* refers to awareness that the violent hookup deteriorated the physical and mental health of the victim. *Consciousness of coerced forgetting* means the person’s consciousness of *coerced forgetting*, that is, of distorting details of the relationship as a result of the pressure received from the dominant coercive discourse *Recalling egalitarian men*, *their value, and attractiveness* means the recall of men in the subject’s biography that had an opposite profile to the harasser and therefore were egalitarian and attractive. *Consciousness of devaluation* implies the subject’s awareness that her value in terms of attractiveness went down because of having had a disdainful hookup. Finally, *incoherence* refers to behaving in ways that go against the values that the subject proclaims to defend.

Following the communicative methodology of research, the data analysis was not limited to the list of theory-informed categories discussed with the participant, but enough room was left in the analysis to identify in the data new themes that were relevant to the participant’s experience. Thus, the data were analyzed in relation to both the list of initial categories and the themes that the subject raised as relevant in discussions. For each final category and theme, content analysis of the data shared in the diary and the interview was performed. Consensus for the coding was achieved through discussion among the researchers. Discrepancies were resolved primarily by clarifying the interpretation of fragments of the written memories in relation to the definition of the categories, and the emerging themes were analyzed. Qualitative data analysis software was not employed.

## 3. Results

The analysis of the memories started with one memory before Simonetta had the hookup that we analyze in depth here, which is the fourth, with a man named Orsino. The previous hookups were also disdainful but involved only kissing without any touching. This fourth included physical violence in touching her. She never again had any disdainful hookups.

Since we began the analysis with that prior memory, we numbered the year of that recall as year 1 (See Table 1). The hookup that is the main focus of analysis in this research also occurred in year 1. In the first memory analyzed, Simonetta referred in her diary to being sad and not feeling pretty. Her former boyfriend, Emilio, had left her and ignored her. In that first recall, Simonetta mentions another previous hookup in the disco Grease, after her boyfriend left her, and she recalls that the boy in this hookup, Giacomo, put her down constantly. Weeks later, Simonetta felt even less attractive and sad:

I’m sick of being sad and not feeling pretty. Emilio has not only left me, but he won’t even look at me. The night of Grease at least I felt triumphant for a while, even though Giacomo talked badly about me everywhere afterwards. But tomorrow we are going to rock again in Grease. The night won’t end without someone wanting to hit on me.

The next day, as she had planned, is when the episode of the disdainful hookup that we analyze in depth took place: in the same disco, Grease.

### 3.1. Recall 1: Disdainful Hookups

In the interview, Simonetta explained that a year and four months after the fourth hookup, the one with Orsino, she started reading scientific literature and engaging in conversations about what boys in disdainful hookups say about the girls with whom they have such relationships. What the literature [28] has shown is that such men speak of these women in very humiliating ways, e.g., as “easy women”. After getting to know this, Simonetta decided not to have more of those hookups:

Soon after, I started reading and commenting on scientific literature about what those guys say about the girls they’ve had those kinds of hookups with. I decided not to have any more [of those hookups] and was glad I had fewer and less bad hookups than all my girlfriends.

Afterwards, Simonetta recalled the hookup with Orsino and wrote it down. On this occasion, she recalled that it was a very short hookup and that she neither knew anything about Orsino, nor did she want to know about him. The same happened to Orsino. They would not recognize each other afterwards:

My fourth punctual hook up didn’t even last 10 min, it started dancing at the Grease nightclub and ended outside. I don’t know anything, and I don’t want to know anything about that guy, and he doesn’t want to know anything about me. Even if we met again, we wouldn’t even recognize each other.

### 3.2. Recall 2: Forced Sex

On the 6th year and 7 months after the violent hookup with Orsino, Simonetta read and had conversations about scientific literature on sexual consent [29] and came to know that there was no consent in the hookup she had with Orsino and that the boy forced Simonetta:

I started reading and commenting on scientific literature on consent and discovered that there had been no consent in that case. Is clear that I consented to him kissing me on the mouth, but he forced me to touch me. He used physical force to touch me.

Then, Simonetta recalled the episode for the second time. This time she recalled details that informed the absence of sexual consent; it was forced sex:

That fourth hookup was the worst. From the beginning, he squeezed me against a wall and kept groping me until he told me to fuck, I said no, I left leaving him standing up and I was glad that we didn’t even know who we were.

### 3.3. Recall 3: Consciousness of Disdain and of Coerced Forgetting

In the 10th month of the 7th year after the disdainful hookup with Orsino, Simonetta read and was involved in conversations about the disdain with which the boys in violent sporadic relationships treat women and talk about them. Such scientific literature [28] has indicated that on most occasions, such men insult women with whom they have had hookups and humiliate them and even their children. In the interview, Simonetta explained that after knowing that evidence, she went online to look up messages on the disco’s website, and she corroborated such disdain:

I read scientific literature and participated in debates on it, about the disdain with which these guys treat girls who have been subjected to them in sporadic hookups, frequently [that disdain] during the girl’s entire life. I didn’t want to believe it and looked it up on internet forums. I saw that there were a lot of posts about the Grease nightclub made by guys who claimed to hook up there and they all called ‘sluts’ the girls who had a hook up there.

At that time, Simonetta wrote again the memory of her third and fourth hookups at Grease, the one with Giacomo and the one with Orsino. She recalled many details that showed that much disdain that was present in both episodes. For instance, she recalled that after the hookup with Giacomo, he disseminated all around her city that she was a whore. As evidence, she shared that, some days later, her neighbor approached Simonetta and mentioned to her the hookup with Giacomo, with a tone and gesture that was very informative of the despising language he was using to talk about her widely, while there was also disdain in the way he talked to Simonetta:

The third casual hook up with Giacomo, which I had in that same nightclub, spread the word throughout my city that I was a slut and that he didn’t want to see me again. A neighbour of mine, shortly after, told me about this affair that he had heard about in a way that I didn’t like at all.

Importantly, the written memory in this third recall included new relevant details about the fourth hookup. Simonetta recalled that Orsino and she had a friend in common and talked about him. In fact, this man in common was a classmate of Simonetta. She reports that this classmate and people of the same context that they shared changed their attitude toward her the days after the hookup. Simonetta also reported awareness that she had wanted to forget that she did know the boy in the fourth hookup, Orsino. Likewise, she acknowledged that she had also wanted to forget that there were people watching them having the hookup outside the disco. She came to recall that, because of this awareness, the next day, she was obsessed with searching for any picture of them that could had been published on the disco’s website:

While he [Orsino] was taking me out of the disco, we talked about a friend of his who went to class with me. I see now that I wanted to forget that I did know the guy from that fourth hookup, and that the common friend and other people around him changed their attitude a lot with me in the next few days. It’s also not true as I said and thought before that no one had seen us; I was crushed against the wall a few meters from the exit, and it was full of people. The next day, I obsessively looked at the photos of the disco in case they had taken one of me. In one of them I found the boy [Orsino] with his friends, and I recognized him perfectly. I see very clearly that he also knows who I am.

### 3.4. Recall 4: Consciousness of Absence of Pleasure and Shame

Two days after the previous recall, in the 10th month of the 7th year, Simonetta started reading and engaging in conversations about scientific evidence on how memory sharing and reconstruction could alleviate the suffering of sexual abuse victimization [30]. Simonetta explained in the interview that, suddenly, with such scientific evidence, she could see the episode of the fourth hookup with Orsino with all the details and as it really was:

I was also reading and commenting on scientific literature about how memory reconstruction helped many girls to free themselves from the heavy burden of having suffered child abuse, adult harassment, rape, etc. Suddenly, the events as they had happened opened to me and I found concrete evidence of them in conversations and writings I had had in the following days after the hookup.

Simonetta’s fourth recall of the hookup with Orsino included new details that informed about her consciousness of absence of pleasure, forced sex, and consciousness of memory falsification. In her diary, Simonetta wrote that the hookup was very painful physically, that it was horrible, and that she did not like it. Simonetta recalled very specific details of the boy forcing her, who touched the most intimate parts of her body against her will, and how she used physical force to stop him from proceeding. She also recalled that she was very embarrassed because people in the surroundings could see her intimate parts, take pictures, and make videos of it. Simonetta also recalled that she was also ashamed because no one had ever touched her vagina, including her ex-boyfriend. She also reported in her diary that after reading the literature and recalling these violent details, she searched for judgments of similar cases and realized that they had been condemned as rape:

It hurt me a lot and I told this to Carlota, the only girl I talked to about it. I also told her that it was horrible, that I didn’t like it at all and that I was disgusted by that guy. As soon as Orsino smashed me against the wall, I tried to move away, but I had no room, I closed my legs very tightly because he was touching my pubis and trying to put his finger inside me; he could not do that because I managed to squeeze my legs with more physical strength than his hand was exerting on me, but he did get to touch my pubis without getting inside me. I was terribly embarrassed because the people around me could be taking pictures or videos, because no one had ever touched me there, not even my boyfriend for a long time, and even because I was ashamed to be seen that my pubis was not well shaved. I read other things besides scientific literature and even sentences showing that similar cases had been convicted of rape, since rape does not require penetration.

### 3.5. Recall 5: Peer Coercion by Young Women and Enslaving Oneself to It

A month after recall six, in the 11th month of the 7th year, Simonetta read about and was involved in conversations on the coercive dominant discourse [31], which pressures young women to have sporadic sexual-affective relationships with boys who have violent attitudes and behaviors, who humiliate girls. As Simonetta shared in the interview: “I read and commented on scientific literature on coercive discourse”.

After learning about scientific evidence on that topic, Simonetta wrote down a fifth recall of her hookups with Giacomo and Orsino. This time, she recalled the role that her friend Giordana played in Simonetta’s having those hookups. As can be seen in the following revised memory, Giordana was the one who decided that Simonetta had to be “free” after being left by her boyfriend Emilio, with being “free” meaning having hookups. It was Giordana who asked one of the boys in the disco to have a hookup with Simonetta, and it was Giordana who led Simonetta to the second floor of the disco to find someone to have her fourth hookup.

In addition, the details in this recall of Simonetta being subjected to such peer coercion are many. Simonetta recalled that it was Giordana who dressed her up and did her make up for having the hookup with Orsino. Simonetta accepted. And, once on the second floor of the disco, Simonetta asked Giordana if the boy was “handsome”. Simonetta was asking for Giordana’s approval. In the case of the third hookup, the one with Giacomo, Simonetta recalled that, afterwards, she felt obliged to explain to her friends that the hookup was a funny experience. She mentioned in the memory report as being compliant to her friends:

My friend Giordana was the one who decided that, since my boyfriend had just left me, now I could be free and have hook ups. The day of my third hook up, (the first one in a disco), she took me to Grease and it must have been already 5 in the morning when she went to a guy she knew and told him to hook up with me. This one didn’t physically force me like the fourth hook up. Then I felt very despised, and worst of all, I accepted it as normal because I had always heard from Giordana and my friends that it was normal and that nothing was wrong. I felt obliged and submitted to tell those friends, without giving details, how fun that first hook up at Grease had been. I didn’t want to look like a prude or that I don’t know how to have fun. Especially hard for my health was when I was seeing that he had said very dirty things about me that were spreading all around my city, but I did not react against it because in my environment that was considered normal. The day of my fourth hook up, Giordana wanted to come to my house to dress me up in a way that I now see horrible and to fill my lips with lipstick to the point of ridiculousness. She kept telling me how beautiful I looked, knowing that this was what I needed after my boyfriend left me. Around 4 am, she told me to go upstairs to the disco to look for a guy to hook up with. I saw a random guy and asked her if he was cute and she said yes. Minutes later, I was smashed against the wall. It wasn’t just Giordana, that coercive discourse was permeating all around me. After that hook up, I had continuous vomiting, headaches, anguish, and constant crying without knowing why.

### 3.6. Recall 6: Consciousness of Deterioration of Health

Twelve days after the last recall, in the 11th month of the 7th year, Simonetta read and engaged in conversations about the health consequences of disdainful hookups [14]: “I read and commented on the bibliography about the health consequences of one-off roll-ups with these types of guys”. Such evidence has well clarified that such hookups are toxic relationships that worsen physical and mental health [7,8]. Later, Simonetta recalled that the year when she had that hookup with Orsino, she was constantly ill. She recalled having digestive problems, being nervous, feeling ugly, and with no motivation to live. Simonetta lost much weight and had the desire to keep losing more. She recalled that every time she knew that they were going to Grease, before going, she had a physical reaction and felt sick. Simonetta also had stomach pain the days after the night in the disco:

During that year, I kept vomiting and getting sick every now and then. Everything made me feel sick, I was always nervous, I felt ugly, everything seemed heavy, I woke up every morning and I didn’t want to live. I lost a lot of weight even though I was already thin, and I even liked to lose even more. Every time we went to that nightclub, or I knew we were going to go, I would begin to retch, get cold sweat, and start vomiting even before we went. My stomach would hurt like hell for days afterwards.

This recall informs of Simonetta’s awareness of her deterioration of health.

### 3.7. Recall 7: Enslaving Oneself to the Dominant Coercive Discourse, Consciousness of Disdain, and Recalling Egalitarian Men, Their Value and Attractiveness

The next month, in the 12th month of the 7th year after the episode, Simonetta shared with us that she started reading scientific literature on how such disdainful hookups are far from being an exercise of freedom but, on the contrary, are an enslavement to the dominant coercive discourse. She reported that such dominant coercive discourse can make you even forget the worst details of the hookup, making it difficult to have sexual-affective relationships with egalitarian men:

I read and commented on scientific bibliography that these hook ups are not an exercise of freedom but of submission to the dominant discourse. They are so hard that, if there is no memory reconstruction, they subjugate you for life making you forget the worst aspects of the events and making it difficult for you to have free casual hookups and falling in love with guys who instead of despising you, love you, who instead of seeing you as a slut, see you beautiful and tell you so.

When Simonetta recalled the hookup with Orsino afterwards, she remembered that what had happened to her and the degree of being despised in that hookup was very extreme. She wrote that none of her women friends who also had disdainful hookups were exposed that much publicly, and other people she asked who knew more about this kind of disdainful hookup knew nothing of cases like hers. Also, Simonetta recalled with rage that when Orsino forced her, and she tried to stop him, she panted. She recalled that she did it because she was in much pain and wanted to make him believe that she was having pleasure so that he finished as soon as possible. These are all instances of consciousness of disdain, of forced sex, as well as of enslaving herself to the dominant coercive discourse. In this last regard as well, Simonetta recalled that after the third and fourth hookups, she stopped treating the boys that she had liked until that moment well, and she reported to be aware that such boys were better persons and more attractive than the ones in her hookups:

What happened to me is something that now I know happens to those of us who submit ourselves as newbies. The friends I was hanging out with had had a lot of hookups, they saw me as shy, innocent, and reserved. Now I realize that none of them did what they did to me in front of everyone. Today, I know that there are many girls who have hookups like that at the exit of clubs, and they can be seen. Yet I have asked a lot of girls and guys who have gone out a lot at night, and they have never seen a hook up at the exit where everyone gets to see that act. I also looked for stories from other girls about cases like mine and I only got pornographic pages where girls were humiliated. What angers me the most is that even in the middle of the physical fight with the guy in the fourth hook up, there were times when I was panting. I did it because I was in much pain and wanted to make him believe that I liked it so that he stopped as soon as possible. After the first two casual hookups with guys like that, who were studying with me, I stopped treating well the guys who until then I had liked, and they had also liked me. I have recovered many photos and looked a lot on the internet, and I have seen that not only were they better people and I never heard them speak badly about a girl with whom they had had a hook up, but they were also much more attractive.

### 3.8. Recall 8: Peer Coercion by Young Women, Enslaving Oneself to Peer Coercion, Consciousness of Devaluation, and Consciousness of Being Despised

As Simonetta explained in the interview, a day after the last recall (7), she read and engaged in conversations about how those kind of boys do not approach the girls they like in the discos, as they know that such girls are beyond their reach. On the contrary, they approach the girls who they think are less attractive:

I read scientific literature and participated in debates about how that kind of guys in nightclubs do not go for the ones they like because they know they are not within their reach, but for the ones they consider less attractive, because as they have no other options, they think they [these girls] will submit to them.

Later, Simonetta recalled the fourth hookup again, and in her memory report she included details about how she felt those days (non-attractive) and that she was submissive to what her friend Giordana did to her, dressing Simonetta in a way to favor the hookup. This recall included plenty of memories of feelings of devaluation and being despised. Simonetta recalled that when they were in the disco, the boys did not look at them as feeling attracted but as the last option of the night if they ended up without any other girls to hook up with. Simonetta remembered how feeling unattractive made her vulnerable to be submissive to any of those boys with the only objective of seeing herself as chosen by, at least, one of them. She also recalled that while she had that feeling in the beginning of the hookup, very quickly, she felt uglier than ever, and she reports feeling the same now when she recalls the episode:

I remembered how those days I didn’t feel attractive at all, and I let Giordana dress me up and do the make-up on me in a way that made me look very ugly. But she told me that I did look pretty. Inside the disco, I could see that the guys didn’t look at us with faces of being attracted but of seeing us as a last resource in the beginning of the morning if they hadn’t found anything better. As time went by, the fact that we were not chosen even for that, made me feel even less attractive and more willing to submit myself to feeling chosen by at least one of those guys. That’s how I felt at first when he [Orsino] took me out of the disco, but five minutes later, when I escaped from him, I felt much uglier than ever. And I still do when I remember it.

### 3.9. Recall 9: Consciousness of Devaluation and Despise

Two days after recall 8, Simonetta read and had conversations about scientific literature on how some egalitarian men can despise women after they know these women have had disdainful hookups or after hearing inventions about it. She read that when this happens, these men can feel that they have been silly in how they have treated these women before and then get obsessed with accomplishing an “unfinished issue”:

I read scientific literature and engaged in debates about how some of the guys who have been with you and respected you, when they hear not only what you have done with others, but what others say you have done, they feel they have been foolish, and become self-conscious and obsessed with achieving the unfinished business.

That time, Simonetta recalled details about her former boyfriend, Emilio, and how he behaved with her after getting to know from others about Simonetta’s fourth hookup with Orsino at Grease. Simonetta recalled that the people who informed Emilio about her fourth hookup were acquaintances of both. This recall included the detail that, after Simonetta’s former boyfriend got to know about her hookups at Grease, he approached her to start dating again. They did, but that time he behaved very differently as when they had been a couple before. Emilio wanted to have full intercourse with her, for him to be the first one, and he achieved this. Once he did, Emilio left Simonetta. She also recalled how sometime later, at Grease, Emilio approached her to have a hookup. Simonetta recalled that Emilio brought her to the same place where she had the third and fourth hookups, a dirty place on the street, and despised Simonetta in many ways, such as putting the alcohol he had drunk in her mouth while sucking on her. The recall of these details informed Simonetta’s gaining consciousness of devaluation and being despised and also of her former boyfriend after he knew she had disdainful hookups:

I was surprised that my ex-boyfriend Emilio asked me out again, just two weeks after that horrible hook up in Grease. I remembered that the only one I had told then specifically about what that guy [Orsino] had done to me then was the closest friend we had in common with Emilio. I also remembered many other people who already knew about my two hook ups in Grease and also knew him [Emilio]. Two days before the first affair in Grease, I told a friend that Emilio was going to screw himself for leaving me. I was going to screw him by having an affair with someone else. Three days later, we had a big group meeting and, when one of the guys toasted to those who hadn’t spent the weekend alone, Emilio was one of those who said: ‘I haven’t spent it alone’. I thought, ‘well, he’s going to fuck himself’, and I said: ‘I haven’t spent it alone either’. I’m not sure, but I guess I thought that having those two affairs in Grease had an effect, and that I had shown him that other guys liked me.

When we got back together as a couple, I noticed him in a very different way than when we had been together before the hookups. Before my hookups, he hadn’t even tried to touch me down there. Some of my friends, especially Giordana, told me he was taking too long to have sex and I was overwhelmed. Finally, he managed to penetrate me and after doing so he automatically left me again. Sometime later, the whole group went to Grease. Emilio started to make out with me in the same place where the others had done it, in a very dirty street where he would have never done it to me while we were dating. And I would never have accepted it either. He treated me more like the other two guys in the hookups I had there, than like he had done while we were dating. He had a glass of alcohol in his hand, he drank, and he snogged me and poured the alcohol into me. Emilio laughed as I threw up. I realize that those hook ups did not make him consider me more attractive, but both he and I did not see me as a girl to fall in love with but as something else. Now, I see that both the two previous guys and this one walked away from me quickly and feeling as disgusted as when people walk away from garbage once they have left it in the trash bin.

### 3.10. Recall 10: Being Incoherent

A month later, in the first month of the 8th year, Simonetta read and engaged in conversations around scientific literature on how disdainful hookups are contrary to the democratic and progressive values that most say to defend, including herself. In the interview, Simonetta expressed this as follows:

I read scientific literature and participated in a debate on how this type of punctual hookup goes against all our values, against our ethics. We say we are supportive, and criticize as sexist the boyfriends who cheat on our friends, our acquaintances, the ones they leave their girlfriends at home to go hunting for others they consider sluts. However, when we get involved in this kind of hookups, we don’t even think about the fact that we are doing it with this kind of guys, thus participating in the cheating of these other girls, nor do we care if they [these men] are sexist or have raped someone.

This time, Simonetta recalled again her third and fourth hookups. This recall included that she never thought if men like Giacomo and Orsino had a girlfriend, and therefore, due to the hookups with her, they were betraying their girlfriends, or if they were consumers of prostitution. She also recalled that while her female friends had hookups with very chauvinist men, those same girls criticized egalitarian male friends and boyfriends as chauvinist:

I remembered that in none of the two affairs I had in Grease did I care or even think if they were cheating on their girlfriends, if they were rapists, or if they were the type of men who said they first tried to see if they could get a free whore at the disco and, if not, they would then go to pay a prostitute. I had many indicators that they were very sexist. Besides, I knew a long time ago that my friends had those occasional affairs with very sexist guys, while they [the girl friends] criticized as sexist any detail of the best guys with whom we had relationships and even of their boyfriends.

### 3.11. Communicating Vessels: Feeling Attraction toward Egalitarian Men

In the interview, Simonetta explained that after this process of iteration of revision of memories and reconstruction, she had felt progressively more attracted toward egalitarian boys. Particularly, Simonetta shared that this evidence-based reconstruction of memory of disdainful hookups, on the one hand, raised her rejection of boys such as Orsino and Giacomo and increased the feelings of disgust toward them. She looked for the four boys and told them, one by one, that the case of harassment was legally expired, but she would support with her testimony any women who denounced them, and as far as she knows, they changed their misconduct after this conversation. On the other hand, she shared that this process increased her attraction and desire toward egalitarian boys. Today, she is cultivating close relationships grounded on freedom, equity, respect, and love. Simonetta notices this transformation of attraction in many spheres of her life, including her improved wellbeing and increased desire for egalitarian boys:

Since I have reconstructed all these memories, apart from not having those vomiting, stomach aches, dizziness and daily sadness and anguish, I feel more and more attractive and confident. Before my first hookup, I felt attractive and I liked and felt a desire for egalitarian boys. Since my first hookup, and until my first interaction with scientific evidence, I was increasingly having health problems, feeling not attractive, and placing the boys I liked in the “friend zone”, and the worst ones in the “fuck zone”. Since my first reconstruction of memory, I started to feel better, but very slowly. I had never again disdainful hookups, and I even had an egalitarian boyfriend. But the same friends who coerced me to have the hookups, pressured me to get demotivated from my boyfriend. They despised him saying that he was a “good guy”. It was only after I had ruined this relationship that I realized that I had to change a lot to get back to the way I was before the coercive discourse ruined me. It was then that I decided to dive deep into the scientific evidence and discuss it with as many different people as possible. This is how I managed to free myself from the coercive discourse, and return to who I always was. Now, I feel much more capable of inspiring love and falling in love with the type of guys I have always liked, and of being Juliet. Now, I feel desire for them again, and I clearly remember the physical disgust I felt for the guy of the coerced episode, during the episode and later. Now, I get a great feeling from egalitarian guys, of passion and excitement, like the feeling I had before that episode, the most horrible in my life.

## 4. Discussion

The results of our qualitative case study both reiterate findings from the literature regarding traumatic autobiographical memories, as well as advance that research field and the one on gender-based violence in adolescence and young adulthood.

Regarding the field of memory research, Eric Kandel, the Nobel Prize laureate in Physiology or Medicine in 2000, wrote in his *In Search of Memory* [32] book:

But sometimes, horrific memories persist and damage people’s lives, as happens in post-traumatic stress disorder, a condition suffered by some people who have experienced at first hand the terrible events of the Holocaust, of war, rape, or natural disaster.

The case analyzed here shows this well. The memory of the disdainful hookup that Simonetta had with Orsino, a relationship which she came up to recognize as rape according to current scientific developments, damaged her life, starting with her health. After some learning about scientific evidence regarding the negative health influences of toxic relationships [24], our subject shared in her recalls the deterioration of her health after the disdainful hookup. Likewise, the case shows what has been quite demonstrated in the field of autobiographical memory research: that for certain experiences, the key factor for moving into long-term memory is not repetition but the emotional intensity of the personal experience [32]. As shared by Simonetta, she just had four disdainful hookups in her lifetime, so there was not much experimentation of that type of adverse relationship. However, the disdainful hookup with Orsino, which just lasted a few minutes, remained in her mind and body for years.

If we move to the field of autobiographical memory research, our findings also confirm and advance the literature. Memories of sexual-affective relationships are autobiographical memories particularly prominent in adolescence and youth [33]. As autobiographical memories, they are malleable [18]; they can be reconstructed through specific learning experiences and social interactions [19,20]. Our study shows this for the case of memories of disdainful hookups and advances the understanding of how such memories can be reconstructed via learning about scientific evidence related to gender violence in violent sporadic relationships. This process has been defined as *evidence-based reconstruction of memory*.

The subject in our study, Simonetta, read scientific evidence about violent sexual-affective relationships, particularly sporadic ones (i.e., disdainful hookups), the dominant coercive discourse and gender violence victimization, and about the health and social consequences of disdainful hookups. Simonetta also freely engaged in conversations about such evidence. After every reading and discussion, she wrote a new report of her memories of two sporadic sexual-affective relationships, which, given their details, can be qualified as disdainful and violent. The analysis of the memories written in her diary reiterates that autobiographical memories of hookups, such as the ones explored, are malleable in various ways. The ten recalls followed the learning about specific scientific evidence, and the longitudinal analysis of the memories indicates that in every of those recalls, Simonetta remembered details of the disdainful hookups that were related to the scientific evidence she read. There was reconstruction. Eric Kandel [32] stated that simply reading a book can change the anatomy of the brain if we can recall something about the reading afterwards, that is, if we have created a memory. Our results show that the specific scientific evidence read and discussed, which included, among others, the dominant coercive discourse [31], peer group pressure to have disdainful hookups [17], and the characteristics and consequences of disdainful hookups [1], led to Simonetta’s recollection of new semantic and emotional details of the episode that were related to what had been read and discussed.

In so happening, every new recall implied a more extended memory, a memory which included new details of the event aligned with what scientific studies in the field of gender violence victimization have investigated as occurring in violent sporadic sexual-affective relationships [1,15,17]: humiliation, being despised, forced sex, peer group pressure, deterioration of physical and mental health, public scorn and devaluation, etc. And that is the key in our findings. Details that she came to recall, such as panting while being physically forced by the man, is a finding consistent with reactions some women have when experiencing sexual violence to make the aggressor stop as quickly as possible [34].

As the results show, there are many instances of the subsequent recalls where Simonetta was very thoughtful in her analyses of the episode, clearly seeing how the dominant coercive discourse that imposes such hookups works and had worked with her. The initial memories of the hookup excluded details of the relationship that were crucial to see the hookup as an enslavement to the dominant coercive discourse. In this regard, Simonetta was also subject to coerced forgetting, that is, “forgetting” and distorting the episode of the disdainful hookup as a result of the pressure posed by the coercive discourse that, in this case, was also imposed by some of her female friends. Prior research on memories enslaved to the dominant coercive discourse has shown the potential of such memories to influence gender violence victimization [17]. Given this and the prospective functions of autobiographical memories [21,22], we may expect that coerced forgetting could increase the likelihood of gender violence victimization in hookups. This is a line of research that could be further explored in bigger samples.

In addition, research in the field of autobiographical memory has shown that one can look at the past and remember it differently depending on very different factors, such as particular emotional states, developmental periods, objectives of the self, and important life experiences [33]. Our results expand those findings in showing, for the field of sexual-affective relationships, that such a look at the past can be deformed by the coercive discourse via coerced forgetting. Additionally, for the case shared, such a deformed look could be transformed via learning about the scientific evidence on preventive socialization of gender violence.

## 5. Conclusions and Practical Implications

The qualitative case study reported in this article illustrates the process of evidence-based memory reconstruction and does so in relation to autobiographical memories of a social problem that has become of much concern worldwide: gender violence victimization among young women. Regarding our findings, the process of learning about scientific evidence on gender violence victimization in sporadic relationships, discussing them, and then revising memories of such violent relationships is promising for preventing gender violence victimization among adolescents and young women. The analysis of this process can also aid exploring risk behaviors in adolescents’ and young adults’ sexual-affective relationships. This exploration can not only be useful for the researchers but also for the adolescents and young adults themselves, who can gain greater awareness of the quality of their intimate relationships and make decisions accordingly that can improve their social relationships and health.

In our case study, beyond achieving a richer memory, what is of the most relevance is that the progressive recalling of more and more details related to the learned scientific evidence led Simonetta to higher consciousness about the very violent character of the hookup, about the absence of pleasure but the experience of harm, about the extreme pressure from one of her friends to have the disdainful hookup, and even about the awareness that such a hookup was rape.

The greater awareness and willingness to break free from the chains of the dominant coercive discourse supported Simonetta’s decision to end the association between attraction and men with aggressive attitudes and behaviors and not only at the cognitive level but at the emotional one, too, which is crucial. In other words, through evidence-based reconstruction of memory, she could dismantle the coercive discourse in her life, break from it, and free her sexual-affective desire. This last aspect is evidenced in the last part of the interview with her, where she reports that, while reconstructing the memory of the event accompanied by the readings and conversations, she progressively felt more and more attraction toward egalitarian boys. This outcome in attraction has to do with the prospective functions of autobiographical memories.

The memory changesIced by Simonetta and associated with a questioning of the dominant coercive discourse supported a projection of a future not only free from disdainful hookups but full of freed desire. This is aligned with other research [14] but goes beyond in showing that such free and healthy projections can result from learning about scientific evidence on disdainful hookups. Furthermore, the results of this case study indicate that such evidence-based reconstruction of memory translated for Simonetta into new emotions, feelings, and even new transformative behavior.

Future studies could further explore these results with larger and more diverse samples. The development of this line of research can have important implications for the prevention of gender-based violence victimization among young women, which mostly occurs in sporadic relationships or hookups. The approach of evidence-based memory reconstruction could be included in therapeutic and social work settings and in programs addressed to prevent gender violence among youth. In this last regard, considering the influence that peer social relationships have for sexual-affective relationship development, the evidence-based memory reconstruction approach could also be promising in peer group interventions. All of these constitute research questions for further research that could have a relevant social impact on young people’s lives.

## Figures and Tables

**Table 1:** Chronology of the episode and the subsequent recalls.

Episode and Subsequent Recalls	Year and Month
Episode of the fourth hookup	0
1st recall	1 year and 4 months after the episode
2nd recall	6 years and 7 months after the episode
3rd recall	7 years and 10 months after the episode
4th recall	7 years and 10 months after the episode
5th recall	7 years and 11 months after the episode
6th recall	7 years and 11 months after the episode
7th recall	7 years and 12 months after the episode
8th recall	7 years and 12 months after the episode
9th recall	7 years and 12 months after the episode
10th recall	8 years and 1 months after the episode

## Data Availability

The original contributions presented in the study are included in the article, and further inquiries can be directed to the corresponding author/s.
